# Gait adaptations on a treadmill in the moderate exercise intensity domain – Comparison between older adults with and without a history of falls

**DOI:** 10.1371/journal.pone.0344711

**Published:** 2026-03-12

**Authors:** Kyra Woitzik, Arber Gashi, Tania Zieschang, Jessica Koschate-Storm

**Affiliations:** Department for Health Services Research, Geriatric Medicine, Carl von Ossietzky University, Oldenburg, Germany; Pennsylvania State University Main Campus: The Pennsylvania State University - University Park Campus, UNITED STATES OF AMERICA

## Abstract

**Objective:**

Older adults face the major risk of falls, potentially resulting in severe functional impairment or death. Many fall scenarios involve components of walking, which is the most common activity in daily life. This study investigated gait adaptations in older adults with and without a history of falls during moderate exercise intensities on a treadmill. The aim was to determine if differences in gait parameters, particularly step length (SL), become more pronounced throughout an interval at moderate exercise intensity, and whether such differences might reflect gait adaptions associated with an elevated fall risk.

**Methods:**

A total of 87 participants were included, of whom 44 had experienced a fall event within the past 12 months or a severe fall within the past five years that resulted in hospitalization or a fracture. Spatiotemporal gait parameters were recorded on a treadmill during preferred walking speed (PWS), 50% PWS, and at different intervals of a six-minute walking trial at the first ventilatory threshold (VT1), resulting in seven distinct intervals that were selected for analysis under successive exercise conditions. Analyses of variance were conducted to compare spatiotemporal gait parameters between the groups (with/without fall history) and across the seven intervals.

**Results:**

Individuals with a history of falls showed significantly shorter steps across all intervals compared to those without a fall history (p = 0.007), with the most pronounced differences observed during PWS Recovery (PWS following exertion up to VT1; 5.7 cm) and Start VT1 (first of three consecutive phases of a six-minute walking sequence at VT1; 5.3 cm). On average, they also took wider steps (p = 0.083) across all intervals. There was no interaction effect between interval and group for any of the spatiotemporal gait parameters.

**Conclusion:**

Community-dwelling, physically fit older adults show differences in step length depending on fall history. Differences were most pronounced at the beginning of VT1 exercise (Start VT1) as well as at PWS following VT1 exertion (PWS Recovery). Step length may therefore represent a situational gait characteristic associated with fall history and elevated fall-related concerns, particularly under moderate exercise conditions. Gait adaptions at moderate intensities, especially VT1, may represent relevant targets for preventive interventions aimed at maintaining mobility and safety in older adults.

## Introduction

In the context of Germany’s aging population, with a projected 33% growth in the population above the age of 65 years, and 60% in the population above the age of 80 years by 2060, geriatric care becomes increasingly relevant. [1] Older adults, often suffering from comorbidities, face a major risk of falls, which can result in health problems, such as fractures, head injuries, or lacerations. [2] These incidents can also lead to concerns about falling (CaF), raising the risk of subsequent falls and the development of frailty [3,4], potentially resulting in severe functional impairment or death. [5,6] Maintaining optimal mobility and independence in old age is crucial, especially given that approximately 30% of falls could be prevented through targeted training of balance, strength and gait speed. [7] Many fall scenarios involve components of walking, which is the most common daily life activity [8] and requires adequate dynamic balance. In the aging process physiological changes in gait patterns, including slower gait speed, reduced step length, increased step width, and greater variability in step frequency are observed. [9] Gait speed in older individuals serves as a marker for frailty, falls, fractures, and mortality. [10,11] Gait of older adults seems to be more variable than of younger adults, which is discussed as a potential risk factor for falls. [12,13] Factors such as sarcopenia, multimorbidity, and declining coordinative abilities, caused by age-related neurological changes, contribute to alterations in gait speed and pattern. [9,14]

Kwon et al. [15] found indicators for an asymmetrical gait pattern at individually preferred walking speed (PWS), such as significantly slower gait speed, shorter steps and more variable step times for people who experienced at least one fall event within the past year. Research suggests contrasting effects of physical exhaustion on motor control in older compared with younger adults; a subsequent treadmill test following submaximal to maximal physical exhaustion on a cycle ergometer showed a significant increase in local gait stability for younger adults, whereas older adults showed a significant decrease in local gait stability. [16] Another study showed that minimum foot clearance due to walking-induced fatigue was reduced only in older, but not in younger adults. [17] The studies mentioned, primarily focused on examining this phenomenon after more intense exertion. Building on these findings, our study aimed to investigate whether the gait adaptions become apparent already during a walking bout at intense exertion and to identify the point at which they emerge. Additionally, we sought to explore whether differences exist not only between younger and older adults but also between older adults with and without a history of falls, and whether these differences become more pronounced under moderate exertion levels. Ultimately, our goal was to contribute to the broader understanding of gait parameters that may be relevant for situational gait adaptions and fall-related mechanisms in healthy older adults.

Gait analysis on a treadmill, particularly at moderate intensities like PWS or the first ventilatory threshold (VT1), provides data at an exercise intensity, similar to real-world scenarios and daily life tasks. VT1 represents the beginning of the transition from primarily aerobic to an intensified anaerobic energy supply for muscle contraction [18]. This intensity represents the threshold at which lactate production in the muscles begins to increase, while the body maintains the capacity to clear it efficiently, resulting in relatively stable, but augmented blood lactate concentrations. In older adults, a significant reduction in postural sway, which is associated with improved postural stability [19], has been observed following exercise on a cycle ergometer. [20] The measured blood lactate levels were positively correlated with the velocity of the Center of Pressure (CoP) during quiet standing after exercise. [20] This raises the question whether potential changes in postural stability can also be detected during moderate intensity treadmill walking.

This research endeavor aims to address this gap by investigating gait speed, step length (SL), step width (SW), and their variability, with a particular focus on disparities between individuals with and without a history of falls. We expect to identify shorter step lengths and increased gait variability in individuals with a history of falls compared to those without, particularly under increased exercise intensities, such as VT1. Furthermore, we hypothesize that these differences will become more pronounced as exertion progresses, providing insight into how gait characteristics associated with fall history evolve under increasing physiological demand and thereby contributing to future efforts aimed at identifying potential thresholds for early fall-related gait changes. Closing this research gap will contribute to a more comprehensive understanding of the fundamentals of gait alterations in the aging population, facilitating the development of effective strategies to enhance mobility.

## Materials and methods

### Participants

Data were derived from the study ’Cardiorespiratory Fitness – Influencing factor for fall risk and gait safety in older people‘ (CareFall), an explorative approach with the primary aim to investigate a potential linkage between individual aerobic fitness and risk of falling. The study, whose secondary outcomes are presented here, was approved by the Medical Ethics Committee of the Carl von Ossietzky University Oldenburg, in Oldenburg, Germany [2021−026] and is registered at the German Clinical Trials Register under the ID DRKS00024890. Participants were recruited primarily through newspaper advertisements, verbal recommendation through other participants, flyers, and newspaper advertisements in Oldenburg, as well as via the homepage of the Division of Geriatric Medicine at the University of Oldenburg. All participants gave their written informed consent prior to the study. Baseline data were collected from 03/03/2022 to 07/31/2023.

The inclusion criteria comprised: 1) minimum age of 65 years; 2) patients without a history of falling; 3) patients, that had either suffered at least one fall event within the last 12 months, or had sustained fractures due to a fall, or had been treated in a hospital due to a fall within the past five years. Exclusion criteria were defined as: 1) diseases that rule out a performance test in the moderate exercise range on a treadmill or acutely impair gait (severe orthopedic, neurological, cardiovascular, pulmonary, metabolic, and visual impairment); 2) body weight > 135 kg for technical reasons; 3) incapacity to walk for 30 minutes without a walking aid; 4) incapacity to give consent to participate in the study.

Participants were stratified into two groups according to their individual fall history. For data processing, each participant was given a 6-digit random ID to be entered into the REDCap data management system [21], which allowed a pseudonymized evaluation of the data.

### Study design

**Fig 1:**
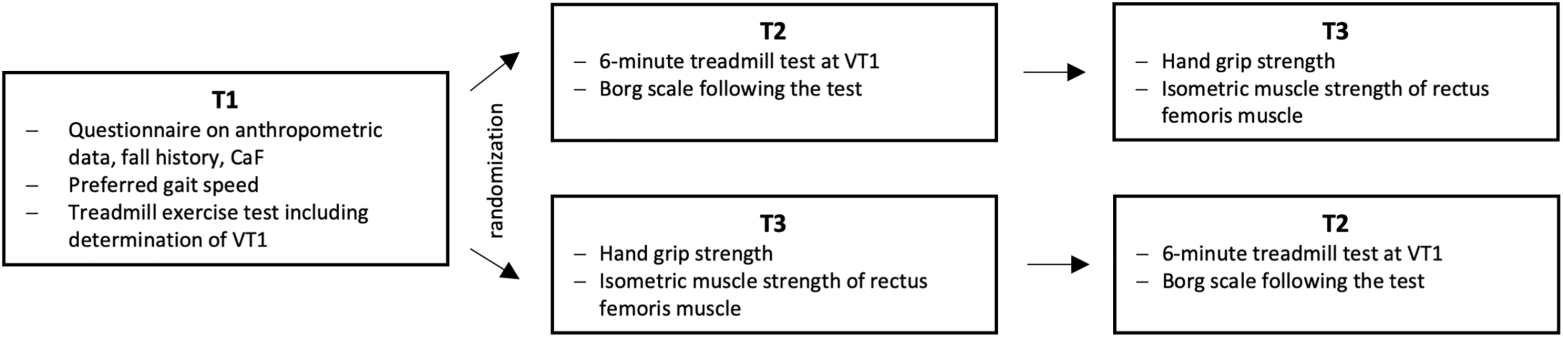
Study design. T1: first appointment, T2: second appointment, T3: third appointment, VT1: first ventilatory threshold.

The tests were conducted at the gait laboratory of the Division of Geriatric Medicine at the Carl von Ossietzky University Oldenburg. Study participants performed physical performance tests and answered questionnaires at three different visits, as shown in Fig 1. Time between visits was set to a minimum of seven and a maximum of 14 days.

During the **first visit (T1)**, baseline characteristics were obtained: epidemiological data, history of falls and CaF, using the Short Falls Efficacy Scale – International (Short FES-I) [22]. Static balance during standing was assessed via a lumbar-placed inertial measurement unit (IMU; Opal V1, Mobility Lab™, APDM, Inc., Portland, OR, USA). The kinetics of the cardiorespiratory system as well as VT1 were examined during an exercise test on the M-Gait treadmill (Motek, Groningen, Netherlands). The treadmill featured a split belt with dynamic adjustments and separate 3D force measurements, with a force-measuring plate installed on each belt. The treadmill protocol to assess cardiorespiratory kinetics and VT1 was individually adjusted using the pre-assessed PWS and a stepwise increasing protocol with individual speed and incline, as suggested by Porszasz et al. [23], to apply a linear increase in work rate until reaching VT1 as an indicator for the first increase in lactate formation. VT1 was determined, according to Beaver et al. [24] using the MetaSoftStudio software of the gas exchange analysis system (MetaMax, Cortex, Leipzig, Germany).

The second and third visits were conducted in random order to avoid habituation effects to the perturbation treadmill. During one of these visits (**T2/T3**), each participant completed a six-minute treadmill exercise at a speed corresponding to individual VT1, performed either at the second or the third visit according to randomization. A short period of 30 seconds at PWS (Pre-VT1 PWS) preceded the exercise phase. Subjective rating of perceived exertion (RPE, Borg Scale) was assessed 15 seconds prior to the termination of the exercise phase at VT1.

During the other visit (**T2/T3**), maximum isometric strength of the rectus femoris muscle of the dominant leg was assessed with participants seated at 90° knee flexion and instructed to perform a maximal isometric extension against an ancle cuff connected to a force transducer until a force plateau was reached (GSVmulti V1.48, ME-Meßsyteme GmbH, Hennigsdorf, Germany. In addition, average hand grip strength was obtained over three measurements for both the right and left hand using a hand dynamometer (DHD-1, Saehan Corporation, Belgium), along with other assessments from which the data are not reported in this research paper. The dominant side (right/left) was determined based on the participants’ self-reports. Five participants who had been retrained from left- to right-handedness during childhood were assigned to the right-handed group.

### Data analyses

To determine gait parameters, the Center of Pressure (CoP) was calculated from the combined vertical forces of both treadmill belts, recorded by the embedded force plates (M-Gait, Motek, Amsterdam, Netherlands) at 300 Hz. CoP trajectories in the anteroposterior and mediolateral directions were used to detect initial contacts, identified when the gradient of the CoP signal crossed a near-zero threshold, indicating contralateral foot strike. Step length was calculated as the belt displacement between two consecutive initial contacts, while step width was defined as the mediolateral distance of the CoP at consecutive initial contacts.

For preprocessing, force plate data were first low-pass filtered with a second-order Butterworth filter at 0.1 Hz. Outliers in the vertical force signal were corrected by linear interpolation using a moving median with a window width of 4 samples. The signals from both plates were then combined using weighted averaging, followed by a second-order Butterworth low-pass filter with a cutoff frequency of 4 Hz. These procedures ensured reliable CoP trajectories, which were subsequently used to calculate spatiotemporal gait parameters. All steps were implemented in Matlab® (R2022a, Natick, USA) using an algorithm developed at the Division of Geriatric Medicine.

Gait parameters included averages and standard deviation (SD) of step length, step width, speed, as well as incline of the treadmill. To provide a second measure of gait variability in addition to SD, Coefficient of Variation (CoV) was calculated according to Taborri et al. [25], using the corresponding formula *CoV = SD/ mean x 100*. The CoV is used to normalize the dispersion relative to the mean, allowing for comparison of variability across different gait parameters. Additionally, an index (SL index) was calculated, which normalizes step length relative to walking speed, using the corresponding formula *SL index = step length/ speed*. This approach ensures that differences in step length are not attributable to variations in individual gait speed. To better control for differences in body height, step length was normalized to body height (*SL/ height*) [26] for each interval (see Supporting Information S1 Table).

**Fig 2:**
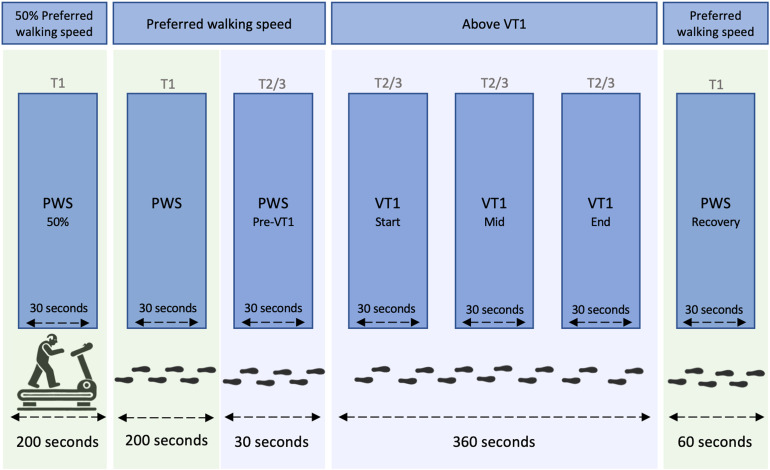
Intervals for gait analysis. Green background marks intervals taken from the ramp protocol test on the first appointment (T1); blue background marks intervals taken from the 6-minute exercise at VT1 (first ventilatory threshold) on the second or third appointment (T2/T3). Intervals did not overlap (Start VT1: 45-75 s, Mid VT1: 165-195 s, End VT1: 285-315 **s)**. For all remaining intervals, analyses were based on the middle 30-second window. PWS: preferred walking speed.

Seven 30-second intervals were then defined and selected for analysis of the gait parameters listed above (see Fig 2). Half of the PWS (50% PWS), PWS preceding participants’ individual ramp protocol (PWS), as well as PWS during recovery (PWS Recovery) were analyzed from the ramp protocol during T1. PWS preceding the 6-minute walking interval at VT1 (Pre-VT1 PWS), as well as three intervals during the individual gait speed at VT1 exercise intensity (Start VT1, Mid VT1, End VT1) were analyzed during T2/3. The 6-minute VT1 trial (total duration 360 s) was divided into three equal phases: Start (0–120 s), Mid (120–240 s), and End (240–360 s). For each phase, as well as for all remaining intervals, analyses were based on the central 30-second window to minimize transient effects and ensure comparability across participants.

### Statistical analyses

Descriptive characteristics were presented as mean ± SD, if a normal distribution could be assumed. Otherwise, the median and 25^th^/75^th^ percentile was reported.

Analyses of variance (ANOVA) were performed with the factors group (with/without a fall event) and interval (50% PWS, PWS, Pre-VT1 PWS, Start VT1, Mid VT1, End VT1, PWS Recovery) for the spatiotemporal gait parameters (step length, SD of step length, CoV of step length, step width, SD of step width, CoV of step width, gait speed, incline of the treadmill, SL Index, SL-to-height ratio). As a sub-question ANOVA were also performed with the group factor CaF (low/moderate-high). Participants who scored ≤8 points on the Short FES-I were classified as having a low degree of CaF, those scoring 9–13 points as moderate CaF, and those scoring ≥14 points as high CaF. Since only one participant fell into the high CaF category, the moderate and high groups were merged into a single elevated (moderate-to-high) CaF group. Sphericity was checked using the Mauchly test. If sphericity could not be assumed, the Greenhouse-Geisser correction was performed accordingly. Post-hoc comparisons were conducted using Bonferroni-adjusted pairwise tests as implemented in SPSS, with adjusted p-values reported directly by the software. [27] The significance level was set at α ≤ 0.05.

RPE, BMI, age and body height were compared between the two groups (with/without a fall history). In addition, maximum isometric strength of the dominant musculus rectus femoris as well as hand grip strength at the dominant side were included as control variables. Lower-limb muscle strength, particularly knee extensor strength, is associated with gait performance and overall functional mobility [28], while hand grip strength is widely recognized as a simple, reliable proxy for general muscular fitness and health status. [29] Accounting for these variables allowed us to control for individual differences in physical fitness that may influence gait characteristics. Both strength measures were entered as absolute values (kg) and were not normalized to body mass. Independent t-tests were applied if a normal distribution could be assumed; otherwise, a Mann-Whitney-U-test was used. If normal distribution could be assumed, exploratory correlation analyses were performed, using the Pearson correlation coefficient, otherwise, the Spearman correlation coefficient was applied. Sex and CaF-groups (low/moderate-high) were compared between the groups (with/without a fall history) using Pearson Chi-Square test.

Statistical analyses were conducted, using SPSS® Statistics, version 29 (IBM, New York, USA).

## Results

### Participants’ characteristics

Anthropometric data of the study population (n = 87) is presented in Table 1. Of the total 105 participants, 18 participants were excluded from further analyses due to unclear label assignments of the export of the treadmill protocol from the treadmill software (n = 7) or due to technical issues with data synchronization (n = 11).

**Table 1:** Study population.

	All participants (n = 87)	With fall history (n = 44)	Without fall history (n = 43)	Significance (two-tailed)
**Age** (years)				p = 0.919
Mean	72.99	73.05	72.93	
SD	± 5.26	± 6.01	± 4.44	
Min. - Max.	65 - 86	65 - 86	66 - 82	
**Sex**				p = 0.160
Male	34	14	20	
Female	53	30	23	
**Body-Mass-Index** (kg·m^-2^)				p = 0.968
Mean	24.43	24.42	24.45	
SD	± 2.90	± 2.87	± 2.95	
Min. - Max.	18.42 - 33.48	18.42 - 31.08	18.94 - 33.48	
**Body height** (m)				**p = 0.011**
Mean	1.72	1.69	1.74	
SD	± 0.09	± 0.09	± 0.10	
Min. - Max.	1.55 - 1.92	1.55 - 1.92	1.57 - 1.92	
**Body mass** (kg)				p = 0.110
Mean	72.53	70.39	74.71	
SD	± 12.63	± 12.25	± 12.78	
Min. - Max.	50.01 - 112.49	50.01 - 103.40	51.99 - 112.49	
**Gait speed at VT1** (m·s^-1^)				p = 0.065
Mean	1.32	1.28	1.36	
SD	± 0.20	± 0.20	± 0.19	
Min. - Max.	0.89 - 1.78	0.89 - 1.28	0.94 - 1.78	
**Incline at VT1** (%)				p = 0.589
Mean	3.93	3.93	3.81	
SD	± 1.07	± 1.07	± 1.01	
Min. - Max.	1.44 - 7.42	1.44 - 6.00	2.18 - 7.42	
**Concerns about falling** (score in Short FES-I)				**p = 0.003**
Mean	8.53	9.11	7.93	
SD	± 1.63	± 1.83	± 1.14	
Min. - Max.	7 - 15	7 - 15	7 - 11	

Abbreviations: SD: standard deviation, Min: minimum, Max: maximum, PWS: preferred walking speed, VT1: first ventilatory threshold, Short FES-I: Short Falls Efficacy Scale-International.

Participants with a history of falls were of significantly smaller stature (p = 0.011) and reported a significantly higher CaF score (p = 0.003; 19 participants with low, 24 with moderate, and one with high CaF) compared with participants without a history of falls (32 with low, 11 with moderate, and none with high CaF). Apart from this, there was no significant group difference for the other categories in terms of fall history.

As shown in Table 2, there was no significant difference between the groups regarding maximum strength of the rectus femoris muscle of the dominant leg (p = 0.557), or in the maximum hand grip strength of the dominant hand (p = 0.180). While not statistically significant, the strength values tended to be lower in the group with a fall history across all measured parameters.

**Table 2:** Force measurements based on fall history.

Force Measurement	Fall History	Mean ± SD Min. - Max.	Significance (two-tailed)
**Maximum strength of the dominant M. rectus femoris**	with fall history	35.46 ± 12.31 15.99 - 74.06	p = 0.557
[kg]	without fall history	36.92 ± 10.66 16.75 - 63.60	
**Maximum hand grip strength of the dominant hand**	with fall history	31.01 ± 8.87 17.70 - 49.90	p = 0.180
[kg]	without fall history	33.78 ± 10.23 16.30 - 66.10	

Abbreviations: SD: standard deviation, Min: minimum, Max: maximum.

### Gait parameters stratified by history of falls

All detailed gait parameters, stratified by individual fall history, are presented in Table 3. For each gait characteristic, a significant main effect of interval was identified (p < 0.001), whereas no significant interaction between group and interval was observed. Step length was the only parameter to show a significant main effect of group. Participants with a history of falls exhibited significantly shorter steps compared with those without a fall history across all seven intervals, with significant between-group differences observed at each interval (50% PWS: p = 0.021; PWS: p = 0.005; Pre-VT1 PWS: p = 0.019; Start VT1: p = 0.007; Mid VT1: p = 0.025; End VT1: p = 0.026; PWS Recovery: p = 0.002). Overall, mean step length averaged 57.1 cm [95% CI 54.7 cm – 59.5 cm] in those with a fall history compared with 61.8 cm [95% CI 59.4 cm – 64.2 cm] for those without a fall history. To adjust step length for different gait speeds we implemented the SL index, also presented in Table 3. A trend towards a lower SL index across all intervals was identified for participants with a fall history (p = 0.089). No significant group differences could be identified for mean step width, its SD and CoV as well as SD and CoV of step length.

**Table 3:** Gait characteristics based on fall history across seven intervals.

Gait characteristics	Fall History		50% PWS	PWS	Pre-VT1 PWS	Start VT1	Mid VT1	End VT1	PWS Recovery	Significance main effects
**Step length**	with n = 44	Mean ± SD Min – Max	34.97 ± 7.43* 24.70-44.22	57.36 ± 9.27* 46.99-65.09	54.92 ± 9.10* 44.53-61.11	3.47 ± 9.26* 37.59-74.18	64.67 ± 9.36* 45.49-75.10	65.08 ± 9.30* 45.30-76.36	59.31 ± 8.79* 47.67-68.48	group p = 0.007*
[cm]	without n = 43	Mean ± SD Min – Max	38.78 ± 7.71* 28.70-48.50	62.68 ± 7.79* 52.89-71.87	59.45 ± 8.60* 50.57-66.45	68.72 ± 8.40* 45.44-79.02	68.88 ± 7.74* 43.09-79.65	69.26 ± 7.84* 46.95-80.97	65.05 ± 7.91* 52.9-75.6	**interval p < 0.001** interaction p = 0.397
**SL Index**	with n = 44	Mean ± SD Min – Max	66.91 ± 10.64 39.24-84.46	54.78 ± 4.12 43.79-66.05	52.43 ± 4.10 42.49-61.83	49.80 ± 3.62 41.37-57.12	50.75 ± 3.58 41.04-57.42	51.12 ± 4.10 41.67-59.05	56.73 ± 5.54 47.99-71.00	group p = 0.089
[cm·m^-1^·s^-1^]	without n = 43	Mean ± SD Min – Max	70.31 ± 10.90 52.20-105.38	57.34 ± 6.43 47.73-76.89	53.80 ± 4.51 45.62-63.99	50.82 ± 3.37 45.32-60.61	51.03 ± 3.76 41.10-61.80	51.29 ± 3.67 45.32-60.94	59.48 ± 6.31 48.04-79.05	**interval p < 0.001** interaction p = 0.128
**SD of** **step length**	with n = 44	Mean ± SD Min – Max	4.00 ± 2.10 1.81-12.49	4.49 ± 2.00 1.55-10.86	3.23 ± 1.79 1.31-11.96	5.84 ± 3.01 2.38-14.96	4.9 ± 2.45 2.12-11.97	5.21 ± 2.77 2.39-11.88	3.88 ± 1.94 1.76-11.33	group p = 0.352
[cm]	without n = 43	Mean ± SD Min – Max	4.19 ± 2.22 1.36-11.27	3.84 ± 3.25 1.44-18.26	3.17 ± 1.26 1.66-8.37	5.37 ± 2.81 2.24-14.98	6.23 ± 4.74 2.02-27.59	5.64 ± 3.52 2.01-17.00	4.32 ± 2.34 1.69-14.64	**interval p < 0.001** interaction p = 0.316
**CoV of** **step length**	with n = 44	Mean ± SD Min – Max	11.80 ± 5.84 4.63-29.31	6.26 ± 3.86 2.37-22.50	6.06 ± 3.27 2.59-18.37	9.10 ± 4.05 3.27-19.58	7.55 ± 3.30 3.34-17.65	8.07 ± 4.17 3.42-18.40	6.68 3.45 2.65-18.46	group p = 0.805
[%]	without n = 43	Mean ± SD Min – Max	11.19 ± 6.23 3.36-33.06	6.27 ± 5.65 2.05-31.08	5.44 ± 2.23 2.07-14.34	7.81 ± 3.77 2.69-18.10	8.97 ± 6.34 2.73-37.76	8.08 ± 4.66 3.03-21.03	6.79 ± 3.81 2.39-18.49	**interval p < 0.001** interaction p = 0.409
**Step width**	with n = 44	Mean ± SD Min – Max	16.90 ± 4.77 12.38-21.11	16.94 ± 4.76 10.95-23.51	17.38 ± 4.67 11.92-22.21	17.61 ± 4.61 9.23-23.71	17.37 ± 4.71 9.88-23.56	17.49 ± 4.47 9.13-24.87	17.10 ± 4.92 9.73-24.41	group p = 0.083
[cm]	without n = 43	Mean ± SD Min – Max	14.70 ± 4.09 10.00-18.89	14.83 ± 4.54 8.79-20.55	16.36 ± 4.10 11.84-20.79	16.58 ± 4.06 8.72-21.73	15.87 ± 4.07 7.27-22.06	16.20 ± 3.90 7.77-23.99	15.14 ± 5.36 8.17-21.69	**interval p < 0.001** interaction p = 0.135
**SD of** **step width**	with n = 44	Mean ± SD Min – Max	1.88 ± 0.59 0.99-3.19	2.65 ± 0.77 1.29-4.44	2.06 ± 0.72 1.22-4.98	2.66 ± 0.86 1.09-4.30	2.66 ± 0.81 1.47-4.66	3.13 ± 1.04 1.39-6.42	3.12 ± 0.91 1.13-5.05	group p = 0.595
[cm]	without n = 43	Mean ± SD Min – Max	2.00 ± 0.85 1.08-5.21	2.50 ± 0.66 1.43-3.86	1.86 ± 0.49 1.24-3.33	2.47 ± 0.89 1.07-4.50	2.84 ± 0.98 1.44-6.18	3.20 ± 1.03 1.57-5.45	2.88 ± 0.82 1.02-4.18	**interval p < 0.001** interaction p = 0.200
**CoV of** **step width**	with n = 44	Mean ± SD Min – Max	12.55 ± 6.51 3.94-31.79	17.17 ± 7.90 7.99-38.73	12.66 ± 5.07 5.84-26.10	16.31 ± 6.78 4.96-33.36	16.82 ± 7.88 6.61-42.36	19.61 ± 9.51 6.76-47.00	20.01 ± 8.98 5.34-51.09	group p = 0.478
[%]	without n = 43	Mean ± SD Min – Max	14.81 ± 8.71 6.06-40.38	18.59 ± 8.70 9.69-52.07	12.13 ± 4.51 5.80-26.05	15.47 ± 5.84 6.70-31.68	18.77 ± 7.26 8.77-48.94	20.83 ± 7.88 8.80-36.67	20.97 ± 8.73 6.82-42.42	**interval p < 0.001** interaction p = 0.304
**Gait speed**	with n = 44	Mean ± SD Min – Max	0.53 ± 0.09 0.33-0.74	1.05 ± 0.18 0.66-1.47	1.05 ± 0.18 0.66-1.47	1.28 ± 0.20 0.89-1.75	1.28 ± 0.20 0.89-1.75	1.28 ± 0.20 0.89-1.75	1.05 ± 0.18 0.66-1.47	group p = 0.089
[m·s^-1^]	without n = 43	Mean ± SD Min – Max	0.55 ± 0.10 0.79-0.55	1.11 ± 0.20 0.64-1.58	1.11 ± 0.19 0.69-1.58	1.36 ± 0.19 0.94-1.78	1.36 ± 0.19 0.94-1.78	1.36 ± 0.19 0.94-1.78	1.11 ± 0.20 0.64-1.58	**interval p < 0.001** interaction p = 0.221

Abbreviations: SL: step length, SW: step width, CoV: coefficient of variation, SD: standard deviation, Min: minimum, Max: maximum, PWS: preferred walking speed, VT1: first ventilatory threshold.

A significant main effect of group on step length is indicated by a red star for each interval; post-hoc pairwise group comparisons for individual intervals were significant at p < 0.05.

Seven intervals: 50% PWS {reduced walking speed, taken from the first appointment (T1)}; PWS {PWS after short warmup, taken from T1}; Pre-VT1 PWS {PWS immediately before 6-minute exercise at VT1, taken from the second or third appointment (T2/3)}; Start/Mid/End VT1 {beginning/midpoint/end of 6-minute exercise at VT1 intensity, taken from T2/3}; PWS Recovery {PWS after moderate exertion, taken from T1}.

**Fig 3:**
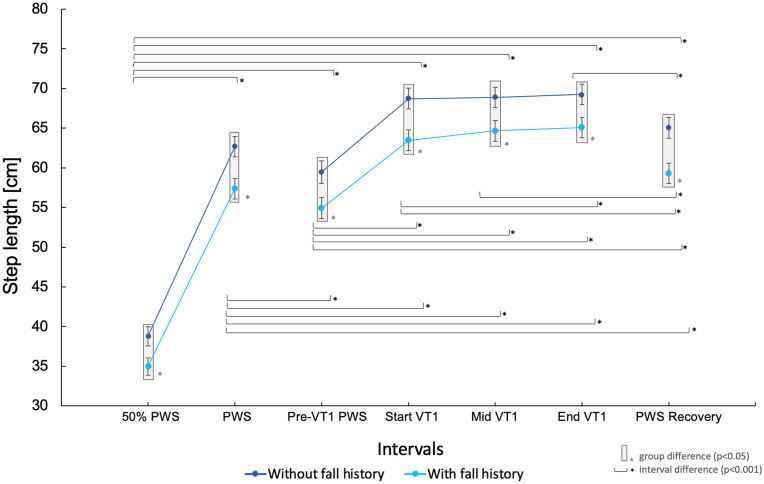
Step length based on fall history across intervals. PWS: preferred walking speed, VT1: first ventilatory threshold.

The maximum group difference (p = 0.002) in step length was detected at PWS Recovery: 5.7 cm [95% CI 2.2 cm – 9.3 cm]. The minimum group difference in step length (p = 0.021) was detected at 50% PWS: 3.8 cm [95% CI 0.6 cm – 7.0 cm]. Fig 3 shows the development of step length across the intervals stratified by fall history.

Because step length is strongly influenced by body height ^[^30^]^, we performed a correlation analysis between these variables to examine whether group differences in step length could be explained by differences in stature. This analysis ensured that the observed effects were not solely attributable to anthropometric variation. Overall, correlation between body height and step length across all seven intervals was statistically significant, with p < 0.001 (see Supporting Information S2 Table). For all participants, Pearson’s correlation coefficients ranged from 0.404 to 0.511.

Body height differed significantly between individuals with and without a history of falls (p = 0.011) and was positively correlated with step length. To account for this, SL-to-height ratio was calculated for each interval (see Supporting Information S1 Table). Based on this adjusted parameter, the group difference remained statistically significant (p = 0.047).

### Gait parameters stratified by CaF (Short FES-I)

In the group with low degree of CaF, 32 individuals had not experienced a history of falls, while 19 did have a fall history. In contrast, among those with elevated CaF, 11 individuals had no history of falls, whereas 25 had experienced one or more falls. All detailed gait parameters based on CaF are presented in Table 4.

**Table 4:** Gait characteristics based on concerns about falling across seven intervals.

Gait characteristics	Concerns about falling		50% PWS	PWS	Pre-VT1 PWS	Start VT1	Mid VT1	End VT1	PWS Recovery	Significance main effects
**Step length**	low n = 51	Mean ± SD Min – Max	38.68 ± 7.48* 28.88-47.95	62.02 ± 8.77* 51.06-71.45	59.40 ± 9.01* 49.98-66.22	68.06 ± 8.37* 43.64-78.54	68.53 ± 7.74* 45.63-79.09	68.89 ± 7.51* 45.85-80.72	64.56 ± 7.76* 53.66-74.59	group p = 0.006*
[cm]	moderate to high n = 36	Mean ± SD Min – Max	34.26 ± 7.52* 24.22-44.57	57.10 ± 8.46* 48.77-64.77	53.99 ± 8.34* 44.09-60.64	63.23 ± 9.64* 37.66-74.01	64.23 ± 9.69* 42.59-75.79	64.67 ± 9.99* 46.59-75.68	58.72 ± 9.16* 45.19-68.61	**interval p < 0.001** interaction p = 0.569
**SL Index**	low n = 51	Mean ± SD Min – Max	68.40 ± 9.28 39.24-86.38	54.97 ± 4.31 43.79-66.38	52.72 ± 4.09 42.49-62.11	50.01 ± 3.16 41.38-56.03	50.43 ± 3.44 41.04-56.55	50.73 ± 3.66 41.67-60.33	57.43 ± 5.32 47.99-71.00	group p = 0.217
[cm·m^-1^·s^-1^]	moderate to high n = 36	Mean ± SD Min – Max	68.86 ± 12.86 44.71-105.38	57.57 ± 6.63 47.74-76.89	53.66 ± 4.66 44.51-63.99	50.72 ± 3.97 44.42-60.61	51.53 ± 3.88 44.46-61.80	51.88 ± 4.11 44.45-60.94	59.02 ± 6.95 49.12-79.05	**interval p < 0.001** interaction p = 0.533
**SD of** **step length**	low n = 51	Mean ± SD Min – Max	3.98 ± 1.98 1.36-11.27	3.96 ± 3.23 1.44-18.26	3.23 ± 1.77 1.65-11.96	5.52 ± 2.90 2.38-14.98	5.64 ± 4.22 2.02-27.59	5.83 ± 3.51 2.01-17.00	4.00 ± 2.14 1.69-14.64	group p = 0.611
[cm]	moderate to high n = 36	Mean ± SD Min – Max	4.35 ± 2.36 1.81-12.49	3.25 ± 1.58 1.56-10.87	3.17 ± 1.15 1.31-6.68	5.72 ± 2.95 2.40-14.96	5.46 ± 3.17 2.35-16.26	4.85 ± 2.48 2.06-11.70	4.24 ± 2.17 1.85-11.33	**interval p < 0.001** interaction p = 0.459
**CoV of** **step length**	low n = 51	Mean ± SD Min – Max	10.78 ± 5.79 3.36-33.10	6.57 ± 5.67 2.05-31.08	5.54 ± 2.97 2.27-18.37	8.08 ± 3.89 2.69-18.10	8.16 ± 5.67 2.73-37.76	8.48 ± 4.92 3.29-21.03	6.21 ± 3.04 2.39-18.50	group p = 0.452
[%]	moderate to high n = 36	Mean ± SD Min – Max	12.52 ± 6.24 5.10-29.31	5.84 ± 3.25 2.37-22.50	6.06 ± 2.56 3.0-13.0	9.00 ± 4.00 4.0-20.0	8.38 ± 4.11 4.0-21.0	7.51 ± 3.51 3.0-16.0	7.48 ± 4.21 4.0-18.0	**interval p < 0.001** interaction p = 0.253
**Step width**	low n = 51	Mean ± SD Min – Max	15.35 ± 4.69 10.87-19.48	15.33 ± 4.97 9.89-21.35	16.32 ± 4.53 11.54-20.87	16.71 ± 4.39 8.69-22.11	16.24 ± 4.62 8.45-21.97	16.60 ± 4.54 7.93-24.20	15.90 ± 5.73 9.20-22.49	group p = 0.294
[cm]	moderate to high n = 36	Mean ± SD Min – Max	16.46 ± 4.34 11.37-20.45	16.70 ± 4.34 10.28-22.74	17.67 ± 4.14 12.06-22.21	17.64 ± 4.30 9.08-23.33	17.17 ± 4.19 8.67-23.81	17.21 ± 3.76 9.20-24.76	16.46 ± 4.43 8.33-23.73	**interval p < 0.001** interaction p = 0.569
**SD of** **step width**	low n = 51	Mean ± SD Min – Max	1.92 ± 0.79 0.99-5.21	2.50 ± 0.71 1.29-4.39	1.93 ± 0.69 1.22-4.99	2.52 ± 0.94 1.07-4.50	2.65 ± 0.87 1.44-6.18	3.19 ± 1.11 1.39-6.42	2.87 ± 0.77 1.24-4.44	group p = 0.323
[cm]	moderate to high n = 36	Mean ± SD Min – Max	1.92 ± 0.63 1.02-3.19	2.69 ± 0.72 1.43-4.44	2.0 ± 0.52 1.28-3.77	2.63 ± 0.79 1.36-4.22	2.90 ± 0.93 1.52-5.75	3.13 ± 0.98 1.57-5.34	3.18 ± 0.98 1.02-5.05	**interval p < 0.001** interaction p = 0.515
**CoV of** **step width**	low n = 51	Mean ± SD Min – Max	14.17 ± 8.30 3.94-40.38	18.43 ± 9.22 8.29-52.07	12.74 ± 5.29 5.80-26.10	15.98 ± 6.58 4.96-33.36	17.75 ± 7.90 6.84-48.94	20.92 ± 9.22 6.76-43.28	22.47 ± 9.45 8.00-51.09	group p = 0.571
[%]	moderate to high n = 36	Mean ± SD Min – Max	12.95 ± 6.86 4.15-31.79	17.09 ± 6.79 7.99-38.48	11.92 ± 4.00 5.85-24.08	15.76 ± 6.00 6.41-28.85	17.83 ± 7.25 6.61-42.35	19.20 ± 7.96 7.65-47.00	20.51 ± 7.98 6.53-44.22	**interval p < 0.001** interaction p = 0.799
**Gait speed**	low n = 51	Mean ± SD Min – Max	0.57 ± 0.09* 0.35-0.79	1.14 ± 0.19* 0.69-1.58	1.13 ± 0.19* 0.69-1.58	1.37 ± 0.19* 0.92-1.78	1.37 ± 0.19* 0.92-1.78	1.37 ± 0.19* 0.92-1.78	1.14 ± 0.19* 0.69-1.58	group p = 0.002*
[m·s^-1^]	moderate to high n = 36	Mean ± SD Min – Max	0.50 ± 0.08* 0.32-0.74	1.00 ± 0.17* 0.64-1.47	1.01 ± 0.16* 0.67-1.47	1.25 ± 0.19* 0.89-1.75	1.25 ± 0.19* 0.89-1.75	1.25 ± 0.19* 0.89-1.75	1.00 ± 0.17* 0.64-1.47	**interval p < 0.001** interaction p = 0.110

Abbreviations: SL: step length, SW: step width, CoV: coefficient of variation, SD: standard deviation, Min: minimum, Max: maximum, PWS: preferred walking speed, VT1: first ventilatory threshold.

A significant main effect of group on step length and gait speed is indicated by a red star for each interval; post-hoc pairwise group comparisons for individual intervals were significant at p < 0.05.

Seven intervals: 50% PWS {reduced walking speed, taken from the first appointment (T1)}; PWS {PWS after short warmup, taken from T1}; Pre-VT1 PWS {PWS immediately before 6-minute exercise at VT1, taken from the second or third appointment (T2/3)}; Start/Mid/End VT1 {beginning/midpoint/end of 6-minute exercise at VT1 intensity, taken from T2/3}; PWS Recovery {PWS after moderate exertion, taken from T1}.

Step length (p = 0.006) and gait speed (p = 0.002) were the only parameters to show a significant group difference regarding CaF, with a significantly slower gait speed (50% PWS: p < 0.001; PWS: p < 0.001; Pre-VT1 PWS: p = 0.002; Start VT1: p = 0.006; Mid VT1: p = 0.006; End VT1: p = 0.006; PWS Recovery: p = 0.001) and significantly shorter step length (50% PWS: p = 0.008; PWS: p = 0.010; Pre-VT1 PWS: p = 0.006; Start VT1: p = 0.015; Mid VT1: p = 0.024; End VT1: p = 0.027; PWS Recovery: p = 0.002) in the group with a moderate to high degree of CaF across all intervals. For each gait characteristic, a significant main effect of interval was identified (p < 0.001), whereas no significant interaction between group and interval was observed.

### Subjective perceived exertion (Borg Scale)

There was no significant difference in RPE after the treadmill test between the groups (p = 0.864). Median was at 14 points on the Borg Scale for both groups of participants (with fall history: IQR: 13–16 points; without a fall history: IQR: 13–15 points).

## Discussion

This study aimed at investigating gait adaptations in older adults with and without a history of falls during moderate exercise intensities on a treadmill. The primary objective was to determine whether the observed differences between individuals with and without fall history would become more pronounced under higher exertion levels, such as VT1, thereby providing insight into how gait characteristics associated with fall history evolve under increasing physiological demand and contributing to future research aimed at the early identification of fall-related gait changes in healthy older adults.

For individuals with a fall history, consistently shorter steps were observed across all intervals compared with those without a fall history. The difference in step length was most pronounced during PWS Recovery (5.7 cm difference, p = 0.002) and during Start VT1 (5.3 cm difference, p = 0.007), suggesting that both, higher exertion levels like VT1 as well as PWS during recovery after VT1 exercise, promote these group differences. Metabolically, a lactate steady state has not yet been reached at Start VT1. Due to the initially insufficient oxygen availability at muscular level, initial lactate levels are higher compared with those after six minutes, assuming a lactate steady state at VT1. [31] Hence, participants without a fall history seem to cope better with slightly increased lactate levels. There is evidence suggesting that higher blood lactate levels, which serve as an indicator for the degree of fatigue, are associated with reduced postural control. [20] Another possible explanation for this group difference is that individuals with a fall history seem to require a period of habituation, during which their step length significantly increases over time from Start VT1 to End VT1 (p < 0.001; see Fig 3). This habituation effect was not observed in individuals without a history of falls. They maintained a consistent step length during VT1, which suggests that step length may be a critical differentiator for older adults with a fall history. Since body height of participants with a history of falls was significantly lower than that of those without a history of falls (p = 0.011), we analyzed the correlation between body height and step length across all seven intervals using Pearson’s correlation coefficient. The results indicated a moderate relationship between body height and step length, with significant positive correlations for each interval (see Table 4). To control for this anthropometric influence, a SL-to-height ratio was calculated for each interval. This normalization allowed for a more standardized comparison of step length across participants of varying stature. Notably, after adjusting for body height, the group difference in step length remained statistically significant (p = 0.047; see S1 Table), suggesting that shorter step length in individuals with a fall history cannot be solely attributed to lower body height. Moreover, the group without a fall history demonstrated lower mean ratios, indicating that they took relatively longer steps in proportion to their height. This may reflect a more confident and dynamic gait pattern.

Physiologically, women tend to be shorter than men. In our convenience sample, there was a higher proportion of women in the fall history group, whereas the sex distribution was more balanced in the group without a history of falls. However, this difference in sex distribution was not statistically significant (p = 0.160). Additionally, slower walking speeds are associated with shorter step length. [32] We calculated an SL index, which normalizes step length relative to walking speed, to ensure that differences in step length are not only attributable to variations in gait speed (see Table 3). A higher SL index indicates that a person takes longer steps relative to their speed. This could suggest that the individual walks more stable or efficiently, as they are able to take larger steps at a given speed. No statistical significance, but a trend toward a lower SL index across all intervals was identified for participants with a history of falls (p = 0.089). This could indicate that these individuals walk less efficiently or may be using compensatory strategies to maintain stability. Shorter steps could be a sign of increased caution while walking, which is more commonly observed in individuals with a history of falls or CaF. [15,33,34]

Although between-group differences in step width did not reach statistical significance, participants with a fall history showed a trend toward larger step width values across all intervals compared to those without a fall history. The wider step width observed in individuals with a fall history may reflect a compensatory strategy to enhance medio-lateral stability, possibly related to impaired postural control, muscular weakness, or CaF. While this adaptation might reduce immediate fall risk, it may also be associated with increased gait variability and reduced walking efficiency, highlighting its potential as both a marker of fall risk and a target for intervention. [35,36] The results of our study align with previously described gait analyses of older adults. [15,33] A systematic review, including 17 studies comparing the gait patterns of individuals with and without a history of falls, with an average age of 77.4 years, found that those with a history of falls tended to have a slower walking speed, shorter stride length, and greater stride width. [33,37]

While statistically significant main effects were identified for intervals for each spatiotemporal gait parameter, no significant effect for the interaction between group and interval was found (see Table 3). This indicates that both intervals and history of falls independently affect gait parameters. Like the stratification of the participants by fall history, significant main effects were shown for intervals but no interaction effects for CaF (see Table 4) were present. Step length was the only spatiotemporal gait parameter that identified participants with elevated CaF, showing shorter steps compared to those with a low degree of CaF. The maximum difference in step length was also observed during PWS Recovery (5.8 cm), suggesting that psychological factors – specifically elevated CaF – may influence gait characteristics to a similar extent as fall history, as both group comparisons revealed comparable reductions in step length. Recent studies have reported similar effects. Kirkwood et al. [34] described how step length appears to be a strong discriminant gait parameter for older women with high CaF. Our results, including both females and males, support this hypothesis. Both CaF and a history of previous falls are considered independent risk factors for future fall events. [2,3] Our findings indicate that a moderate to high degree of CaF have the same impact on step length as history of falls, despite the fact that this group includes 31% of individuals without a fall history. A recently published study highlights the importance of step length in predicting falls among older adults; identifying it as one of the significant factors in a risk prediction model. [38] However, this study recorded gait on a 10-meter walkway. In contrast, we used a different methodology for gait analysis, incorporating a longer duration and higher intensity walking task on a treadmill.

With aging, the adaptive processes that adjust spatiotemporal gait characteristics to maintain optimal walking stability, such as the regulatory capacity of both central and peripheral nervous systems [39,40] become significantly impaired. This leads to declines in both motor and cognitive performance [41], resulting in alterations in step length and stride time. [42,43] Our findings suggest that these mechanisms, which contribute to shorter steps, are further aggravated in older adults with CaF and/or a history of falls. This highlights the importance of fall prevention and suggests that training focused on gait quality could potentially help prevent falls. [44] For instance, targeted training at more intense walking speeds or frequently varying walking speeds under safe conditions, could be implemented to enhance gait safety and stability at those levels where differences in step length and step width are most pronounced. A walking speed of less than 1 m/s indicates motor-postural instability [10], and a minimum speed of 0.8 m/s in daily life should be achieved to ensure good social inclusion and daily participation. [11,45] Setting a focus on gait speed is particularly relevant since slower gait speeds are associated with higher mortality in older adults. [46–48] Although the difference in overall gait speed was not statistically significant in terms of fall history (p = 0.089), which is a prerequisite for the interpretability of its effects on gait parameters, lower PWS were observed among the participants with a history of falls, averaging 1.052 m/s, just above the threshold of 1 m/s. In contrast, participants without a history of falls had a slightly higher average PWS of 1.110 m/s. When participants were stratified according to their CaF, those with a low degree of CaF walked significantly faster than those with elevated CaF (p = 0.002). This observation indicates that individuals with a higher degree of CaF may adopt a more cautious gait pattern, deliberately reducing their walking speed as a strategy to enhance perceived safety and stability, whereas those with lower CaF may tend to walk at a more natural and dynamic pace without fear of losing stability. Regarding gait speeds at VT1, a trend towards lower gait speeds was observed in the group with a fall history (p = 0.065), indicating that this group had a lower level of physical fitness. [49] Reaching VT1 at lower walking speeds suggests that participants with a fall history may have lower aerobic fitness, an earlier onset of fatigue, and consequently, lower mobility and daily functioning. [50–52] This finding raises the idea of implementing balance training, as frequently used for falls prevention training, at PWS on a treadmill following bouts of exercise at VT1 or even more intense exercise intensities, where differences in gait speed were most pronounced.

### Limitations

Due to the cross-sectional design and retrospective assessment of fall history, causal relationships between gait characteristics and fall events cannot be established.

Overall, the study involves a relatively healthy and physically active group of participants, volunteering to take part in the study and presumably with an interest in their own fitness and health. Furthermore, the gait parameters were analyzed on a treadmill. Gait pattern and walking speeds differ between overground and treadmill walking. [53–55] As treadmill speed is often perceived as faster by test subjects compared to the same speed during overground walking [14], preferred walking speed on a treadmill tends to be lower than the speed level of physiological walking on a flat surface. [56] However, a meta-analysis published in 2020 encompassing 33 studies concluded that treadmill walking and overground walking are largely comparable in terms of spatiotemporal gait parameters. [57] It also needs to be taken into consideration that factors other than fall history influence step length. In our study, we attempted to account for these variables as comprehensively as possible. For instance, body height was found to be significantly lower in participants with a history of falls, and we identified a moderate positive correlation between height and step length across all intervals. Although body height was statistically controlled for by calculating the SL-to-height ratio, this approach is a simplification and may not fully account for all influences on gait. Nevertheless, it allows for a more standardized comparison across individuals of varying stature. Additionally, the distribution of women – who are generally shorter than men – was skewed toward the fall history group, potentially affecting the results. By introducing the SL index, which adjusts step length relative to walking speed, we aimed to reduce the confounding effect of gait speed on step length to ensure that variations in step length were not merely a reflection of differences in walking speed. The exercise duration was relatively short, albeit based on previous studies. However, it remains unclear whether different effects would emerge with prolonged exertion.

## Conclusion

This study examines differences in gait characteristics during moderate exercise in older adults and identified step length as an important parameter that differed significantly between participants with and without a history of falls. Although no statistically significant interaction effect was found, the greatest difference in step length between individuals with and without a history of falls, consistent with existing literature, was observed following prolonged periods of walking at higher intensities. Furthermore, the pronounced difference in step length at the beginning of the VT1 phase and during PWS following VT1, when considering fall history and CaF, of the physically fit participants may hold valuable information, which should also be evaluated in less fit participants. These findings may reflect post-fall differences in gait characteristics or underlying risk-related adaptations but do not allow conclusions about temporal causality. Future studies examining gait in the context of fall risk assessment should consider incorporating higher walking speeds in addition to the conventional preferred walking speed conditions to better reveal subtle between-group differences that may remain undetected under low-demand walking conditions, especially in physically fit older adults. However, further research is needed to assess whether such gait changes precede falls and could serve as early indicators.

## Supporting information

S1 Table SL-to-height Ratio based on fall history across seven intervals. (DOCX)

S2 Table Correlation between body height and step length. (DOCX)
