# Supplementary material for: Gait adaptations on a treadmill in the moderate exercise intensity domain – Comparison between older adults with and without a history of falls
Source: PLoS One. 2026 Mar 12;21(3):e0344711. doi: 10.1371/journal.pone.0344711 (PMC12981512; doi:10.1371/journal.pone.0344711)
Supplement: S1 Table — (DOCX) [file pone.0344711.s001.docx]

| Gait characteristics | Fall History |  | **50% PWS** | **PWS** | **Pre-VT1 PWS** | **Start VT1** | **Mid VT1** | **End VT1** | **PWS Recovery** | **Significance**  **main effects** |
| --- | --- | --- | --- | --- | --- | --- | --- | --- | --- | --- |
| **SL-to-Height**  **Ratio [m]** | with | Mean ± SD | 0.21 ± 0.04 | 0.34 ± 0.05 | 0.32 ± 0.05 | 0.37 ± 0.05  p=0.054 | 0.38 ± 0.05 | 0.38 ± 0.05 | 0.35 ± 0.04 | **group p=0.047***  **interval p<0.001**  interaction p=0.453 |
|  | without | Mean ± SD | p=0.073  0.22 ± 0.04 | p=0.031*  0.36 ± 0.04 | p=0.098  0.34 ± 0.05 | 0.39 ± 0.04 | p=0.162  0.39 ± 0.04 | p=0.176  0.40 ± 0.04 | p=0.014*  0.37 ± 0.04 |  |

**Supporting Information 1**. SL-to-Height Ratio based on fall history across seven intervals.

*Abbreviations: SL: step length, SD: standard deviation, PWS: preferred walking speed, VT1: first ventilatory threshold.*

*Post-hoc pairwise group comparisons for individual intervals are shown in boxes and were significant at p<0.05. A significant main effect of group is indicated by a red star for each interval.*

*Seven intervals: 50% PWS {reduced walking speed, taken from the first appointment (T1)}; PWS {PWS after short warmup, taken from T1}; Pre-VT1 PWS {PWS immediately before 6-minute exercise at VT1, taken from the second or third appointment (T2/3)}; Start/Mid/End VT1 {beginning/midpoint/end of 6-minute exercise at VT1 intensity, taken from T2/3}; PWS Recovery {PWS after moderate exertion, taken from T1}.*
