# Supplementary material for: Gait adaptations on a treadmill in the moderate exercise intensity domain – Comparison between older adults with and without a history of falls
Source: PLoS One. 2026 Mar 12;21(3):e0344711. doi: 10.1371/journal.pone.0344711 (PMC12981512; doi:10.1371/journal.pone.0344711)
Supplement: S2 Table — (DOCX) [file pone.0344711.s002.docx]

**Supporting Information 2.** Correlation between body height and step length.

|  | **Pearson’s correlation coefficient between body height and SL** | **All**  **participants**  (n=87) | **With**  **fall history**  (n=44) | **Without**  **fall history**  (n=43) |
| --- | --- | --- | --- | --- |
| **50% PWS** | Coefficient | 0.404 | 0.400 | 0.329 |
|  | Sig. (two-tailed) | **p<0.001** | **p=0.007** | **p=0.031** |
| **PWS** | Coefficient | 0.481 | 0.479 | 0.398 |
|  | Sig. (two-tailed) | **p<0.001** | **p<0.001** | **p=0.008** |
| **Pre-VT1 PWS** | Coefficient | 0.491 | 0.446 | 0.275 |
|  | Sig. (two-tailed) | **p<0.001** | **p=0.002** | **p=0.074** |
| **Start VT1** | Coefficient | 0.483 | 0.408 | 0.475 |
|  | Sig. (two-tailed) | **p<0.001** | **p=0.006** | **p=0.001** |
| **Mid VT1** | Coefficient | 0.511 | 0.459 | 0.509 |
|  | Sig. (two-tailed) | **p<0.001** | **p=0.002** | **p<0.001** |
| **End VT1** | Coefficient | 0.501 | 0.452 | 0.494 |
|  | Sig. (two-tailed) | **p<0.001** | **p=0.002** | **p<0.001** |
| **PWS Recovery** | Coefficient | 0.502 | 0.558 | 0.355 |
|  | Sig. (two-tailed) | **p<0.001** | **p<0.001** | **p=0.020** |

*Pearson’s correlation coefficient between body height and step length (SL) based on fall history across intervals: 50% PWS (preferred walking speed) {reduced walking speed, taken from the first appointment (T1)}; PWS {PWS after short warmup, taken from T1}; Pre-VT1 PWS {PWS immediately before 6-minute exercise at VT1 (first ventilatory threshold), taken from the second or third appointment (T2/3)}; Start/Mid/End VT1 {beginning/midpoint/end of 6-minute exercise at VT1 intensity, taken from T2/3}; PWS Recovery {PWS after moderate exertion, taken from T1}.*
